# OA10.02. Yoga for women with breast cancer undergoing radiotherapy (XRT): a randomized clinical trial with an active stretching control group

**DOI:** 10.1186/1472-6882-12-S1-O38

**Published:** 2012-06-12

**Authors:** L Cohen, K Chandwani, N Raghuram, R Haddad, G Perkins, A Spelman, R Nagarathna, K Johnson, A Fortier, B Arun, Q Wei, C Kirschbaum, H Nagendra

**Affiliations:** 1 The University of Texas MD Anderson Cancer Center, Houston, USA; 2 Swami Vivekananda Yoga Research Foundation, Bangalore, India; 3 Vivekananda Yoga University, Bangalore, India; 4 Vivekananda Yoga Research Foundation, Bangalore, India; 5 Technical University of Dresden, Dresden, Germany

## Purpose

We examined the effects of yoga on buffering changes in QOL and cortisol slope in women with breast cancer undergoing (XRT).

## Methods

Patients with stage 0-III disease were recruited prior to XRT (baseline) and randomized to one of three groups: Yoga (YG-n=53) or Stretching program (STR-n=56) 3 times a week for 6 weeks during XRT or Waitlist Control (WLC-n=54). Self-report measures of fatigue (BFI), depression (CESD), QOL (SF-36), benefit finding (BF), and spirituality (FACT-SP) were completed and saliva collected at baseline, end of treatment, and 1, 3, and 6 months later. We examined change from baseline for questionnaires and slope analyses for cortisol.

## Results

By the end of XRT, the YG and STR groups had a reduction in fatigue while the WLC had an increase (YG: -0.23, STR: -0.45, WLC: 0.52; p’s<.05). At 1, 3, and 6 months after XRT, the YG group had greater increases in SF-36 physical functioning compared to both STR and WLC (1 month: 5.8, 2.0, 0.8; 3 months: 6.5, 3.4, -0.2; 6 months: 6.1, 3.4, 1.1; p’s<.05). The outcomes were similar for SF-36 general health scores. By 3 and 6 months after XRT, there were significant increases in BF for the YG group (3 months: 3.0, -2.6, -2.5; 6 months: 1.1; -3.9; -4.7; p’s<.05). There were no differences for spirituality and depression. Cortisol slope was steepest for the YG group compared to the STR and WLC groups (end of XRT: -0.10, -0.08, -0.08; 1 month: -0.10, -0.09, -0.06; p’s<.01).

## Conclusion

Yoga buffered changes associated with XRT in terms of fatigue, QOL and benefit finding, and resulted in steeper cortisol slopes, while stretching resulted in only modest benefits. This is the first yoga study in oncology to include an active control group, suggesting that the benefits of yoga are due to more than simple stretching or other indirect effects.

